# The Treatment of Pulpless and Gaseous Teeth

**Published:** 1886-07

**Authors:** J. Taft


### The Treatment of Pulpless and Gaseous Teeth.

BY J. TAFT.

(Read before the Mad River Valley Dental Association.)

The precise and proper course to be pursued with teeth, the pulps of which are gone, depends upon several circumstances. For instance, the length of time intervening since the pulp destruction. Second, the agent and influences that have been operating on the tooth since that time. Third, the condition of the parts about the tooth.

The pulps of teeth are destroyed in two or three different ways. First, by undue using of the teeth. Second, by breaking the teeth. Third, by decay. And Fourth, by careless operations upon them.

I presume, however, the question here to be answered is, what shall be done with those teeth the pulps of which have been destroyed before they came to the hands of the dentist?

Some teeth when presented in this condition, seem to be of comparatively healthy condition as far as the tissues about them are concerned; the pulp having died and become disintegrated, and the debris simply removed by washing or some other process, a healthy condition apparently remaining at the end of the root; the crown of the tooth but little or not at all changed in color. The treatment and management of such cases is a very simple matter. Merely the removal of the debris and decaying matter; the appliance of some disinfectant and antiseptic; then letting the canal and pulp chamber be immediately filled with some appropriate material. Various substances have been used for this purpose.

A solution of gutta percha in chloroform, conveyed on fibers of silk, flax, or cotton, is very efficient. Carbolized cat-gut may also be used. Of metals, lead and gold and tin may be used; either of them formed into a wire and driven into the canal or preferably, the gold and tin may be used in the form of foil. Many materials have been used for this purpose. Those here mentioned, however, being perhaps more practicable and more extensively used than any others.

The teeth in less favorable condition than the above, are those, having through the root canal more or less discharge. This will vary greatly in character. Sometimes it is little more than pure plasm poured out from a comparatively healthy living tissue at the end of the root. In other cases, it is the product of a suppurating tissue which presents a more or less offensive ichorious discharge. This will sometimes occur when the tissue about the end of the root is not extensively involved in disease. Sometimes there is in connection with it alveolar abscess. If an abscess is not already formed care should be exercised in the management of the case, lest more disease occurs than was at first found. The discharge of comparatively healthy plasm through a root is from a living surface just under sufficient irritation to prevent healing. This irritation will usually subside upon thorough cleansing of the part, and applying antiseptic preparations. This will be effected in many cases very promptly. In others far more time will be required.

In any case how shall it be known that restoration of the part to a healthy condition has been obtained?

Usually by filling the canal with fibers of silk, flax, or cotton, not too tightly, and hermetically sealing the external opening; permitting it to remain from twenty-four to forty-eight hours, then remove and note if the silk is moistened or has an offensive odor. If it is markedly so, further medication is required. If there is no odor, and the silk is dry, the indications are favorable, and but little risk will be incurred in at once effectually closing the canal, pulp chamber, and cavity of decay. If, however, any fear is entertained in reference to the result, it is quite practicable to make another test during a longer time.

In some cases a discharge that is very offensive at first may be in a very short time greatly changed for the better; and, indeed, in many cases a return to a healthy condition be established within a few days. The agents appropriate for this purpose are iodine and creasote or carbolic acid, peroxide of hydrogen, salicylic acid, iodoform, oil of cloves, etc. A judicious selection and application from this list of agents will usually be quite sufficient, as far as topical treatment is concerned. This is in the first class of cases mentioned where alveoler abscess has not been formed.

There are some teeth from which there is no appreciable escape of fluid, which, if sealed up, cause great annoyance and severe pain. This occurs from vapor or gas products. Such cases are not of frequent occurence, but occasionally are found, and sometimes to the great annoyance of both patient and operator. The operations and treatment are the same as though the product was a fluid. The vapor or gas proceeds from the decomposition of offensive matter that must be removed or neutralized. That being done, the vapor or gas no longer appears and annoys. The treatment after this is the same as the treatment in any case of pulpless teeth.

